# Assessment of *in vitro* efficacy for common surface disinfectants and antiseptics against *Tritrichomonas foetus* trophozoites

**DOI:** 10.3389/fvets.2023.1282274

**Published:** 2023-11-09

**Authors:** Katy A. Martin, Kristofer Kovach, Erica Moscoso, Elizabeth Carreiro, Jeba R. J. Jesudoss Chelladurai, Matthew T. Brewer

**Affiliations:** ^1^Department of Veterinary Pathology, College of Veterinary Medicine, Iowa State University, Ames, IA, United States; ^2^Department of Diagnostic Medicine/Pathobiology, College of Veterinary Medicine, Kansas State University, Manhattan, KS, United States

**Keywords:** trichomonosis, bovine trichomonosis, protozoa, cattle, theriogenology

## Abstract

The protozoan *Tritrichomonas foetus* causes early embryonic death in cattle, there are no legal options for treating this parasite in the United States, and there are few developed protocols for cleaning veterinary and obstetrical equipment that may have been contaminated with trophozoites. In this study, we evaluated bleach, ethanol, acetic acid, chlorhexidine gluconate, and hydrogen peroxide solutions for the ability to kill trophozoites *in vitro*. Our findings suggested that ethanol and bleach could adequately disinfect tools and equipment. Acetic acid, chlorhexidine, and hydrogen peroxide had applications as surface disinfectants in addition to potential as local topical treatments due to their past uses in veterinary theriogenology. Chlorhexidine gluconate demonstrated trophocidal effects by damaging parasite cell membranes and had the lowest effective concentration 50 (EC_50_) of any compound tested and was in the micromolar range. These findings, in conjunction with accepted clinical uses of chlorhexidine gluconate suggest that this is a convenient agent for disinfecting equipment. In addition, topical use of chlorhexidine is relatively common, setting the stage for further investigation of this compound as a topical therapeutic option for bovine trichomonosis.

## Introduction

*Tritrichomonas foetus* is the etiologic agent of bovine trichomonosis, a venereal disease of cattle. Bulls are typically asymptomatic, with trophozoites living on the mucosal surface of the preputial cavity (1). Bulls tend to remain chronic carriers and serve as a source of infection for cows. When a cow is bred by an infected bull, *T. foetus* trophozoites migrate into the uterus via the cervix, colonizing the entire reproductive tract within 2 weeks (2). Infection does not interfere with conception and few lesions are apparent prior to around 50 days of gestation, at which point varying degrees of endometritis, cervicitis, and vaginitis can be observed (3). Most infected cows will lose their pregnancy within the first 5 months of gestation, with losses by day 17 post infection occurring in the majority of cases (1, 4). Ultimately, *T. foetus* infections lead to a reduced calf crop and widespread calving interval which have significant economic impacts for beef cattle producers (5). *Tritrichomonas foetus* persists in North America, including in the United States where it has not been eliminated (6).

While mechanical transmission during mating is the primary route of infection, iatrogenic *T. foetus* infections also occur (7). Evaluation of disinfectant efficacy is important to ensure surfaces, tools, and equipment potentially exposed to trophozoites do not serve as a source of infection. In order to maintain a high level of biosecurity, producers and veterinarians need to have confidence in the disinfectants they choose to utilize. Disinfectants are also important in laboratory settings to maintain aseptic environments for diagnostic and experimental procedures. Agencies have recommended 10% bleach solutions for disinfection of surfaces and equipment for the control of *Trichomonas gallinae* in birds, however, relatively few specific recommendations regarding *T. foetus* are available for the disinfection of tools and equipment.

In this study, five compounds were selected for evaluation of activity against *T. foetus* trophozoites: acetic acid, bleach, chlorhexidine gluconate, ethanol, and hydrogen peroxide. Ethanol and bleach are commonly used surface disinfectants in both laboratory and clinical settings. Acetic acid, chlorhexidine gluconate, and hydrogen peroxide have been studied for efficacy against human STIs, including *Trichomonas vaginalis* (8, 9). The use of disinfectants and antiseptics to treat or aid in the treatment of vaginal infections has several advantages, including low or no resistance to the compounds, reduced side effects compared to systemic antibiotic treatment, potential for prophylactic use, and efficacy in cases of non-specific vaginitis (8, 10–12).

The goal of this study was to evaluate the efficacy of these five compounds against *T. foetus* trophozoites, in order to identify the best candidates for surface disinfection as well as potential candidates for treatment of bovine infections.

## Materials and methods

### Parasites

Two strains of *Tritrichomonas foetus* were used for these experiments, ATTC BP-4 Beltsville strain (ATCC) and an Iowa field strain (IA-1) (13, 14). Trophozoites were maintained in trypticase-yeast extract-maltose (TYM) medium supplemented with 10% adult bovine serum and 1% 100X penicillin–streptomycin (15). Trophozoites were maintained at 35°C and regularly sub-cultured to maintain growth. Cultures were maintained in sterile 15 mL centrifuge tubes filled completely with media and capped tightly to create an anaerobic environment.

### Test compounds

Household bleach (8.25% sodium hypochlorite), acetic acid, chlorhexidine gluconate (2% surgical scrub solution), ethanol, and hydrogen peroxide were analyzed for trophocidal effects in this study. Metronidazole was utilized as a positive control as it continues to demonstrate high efficacy against *T. foetus* (16). Dilutions of test compounds utilized in the assays were prepared in TYM medium and are summarized in Table 1.

**Table 1:** Concentrations of test compounds in this study.

Concentration of killing assay compounds					
Acetic acid	1.0%, 170 mM	0.5%, 80 mM	0.25%, 40 mM	0.13%, 20 mM	0.06%, 10 mM
Bleach	0.8%, 100 mM	0.4%, 50 mM	0.24%, 30 mM	0.08%, 10 mM	0.04%, 5 mM
Chlorhexidine gluconate	0.013%, 140 μM	0.006%, 69 μM	0.003%, 35 μM	0.002%, 17 μM	0.001%, 8.7 μM
Ethanol	30%, 6.5 M	15%, 3.3 M	10%, 2.2 M	3%, 0.65 M	1%, 0.22 M
Hydrogen peroxide	0.13%, 40 mM	0.06%, 20 mM	0.03%, 10 mM	0.02%, 5 mM	0.01%, 2.5 mM

Percent solutions and molarity provided for clarity.

### Killing assays

Assays were carried out in sterile microtubes with starting concentrations of 75,000 trophozoites in 1.5 mL total volume. Test compounds were added in the concentrations provided in Table 1. Assay cultures were maintained at 35°C and BD GasPaks (Becton, Dickinson and Company, Sparks, MD) were utilized to induce anaerobic incubation conditions. Cultures counts were performed using a hemocytometer at 2- and 24-h time points. Experiments were repeated in triplicate using a minimum of two technical replicates.

### Chlorhexidine gluconate recovery assays

Based on the results of killing assays and currently accepted clinical utility in veterinary theriogenology, chlorhexidine gluconate was selected to undergo additional evaluation. The two highest CG concentrations (140 μM/0.013% and 69 μM/0.006%) were used for the recovery assays, along with metronidazole (500 μM) and drug free controls. Trophozoites (75,000) were treated for 24 h in a total volume of 1.5 mL. Following 24 h of treatment, cultures were centrifuged at 2,000 × *g* for 3 min. The supernatant was removed and three washes using 1 mL drug free TYM medium were performed to remove as much of the treatment compounds as possible. The cultures were resuspended in 1.5 mL drug free TYM medium following the final wash. Culture growth was evaluated using a hemocytometer at 24, 48, and 72 h post drug removal. Cultures were maintained at 35°C in a chamber with a BD GasPak to provide an anaerobic environment. These experiments were repeated in triplicate with two technical replicates for each trial.

### *In vitro* membrane permeability assay

For propidium iodide staining, trophozoites were treated with 0.05% chlorhexidine gluconate, 2.5 mg/mL metronidazole, or left untreated for 30 min. Following treatment, trophozoites were stained for 15 min using NucBlue™ and propidium iodide from the Invitrogen ReadyProbes™ Cell Viability Imaging Kit, Blue/Red (Life Technologies Corporation, Eugene, OR).

### Statistical analysis

Graphpad prism statistical software was used to generate kill curves, calculate EC_50_ values, and 95% confidence intervals using a four-parameter logistic regression model.

## Results

### Killing assays

**Figure 1:**
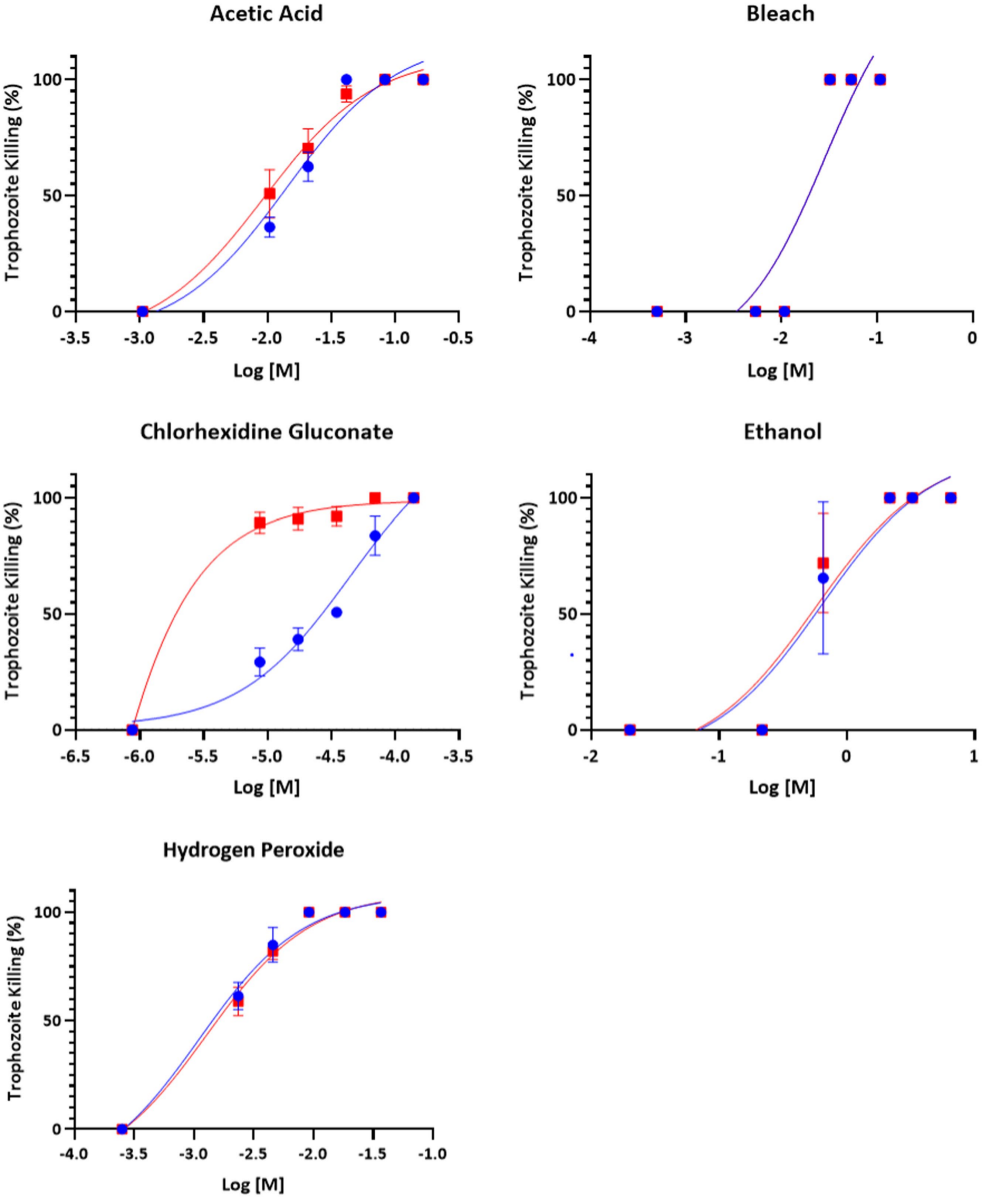
Dose–response relationship of disinfectant compounds against *T. foetus* trophozoites at 2 h *in vitro*. IA-1 trophozoites and ATCC trophozoites are represented by blue circles and red squares, respectively. Points represent mean ± SE. EC 50 values are shown in Table 2.

**Figure 2:**
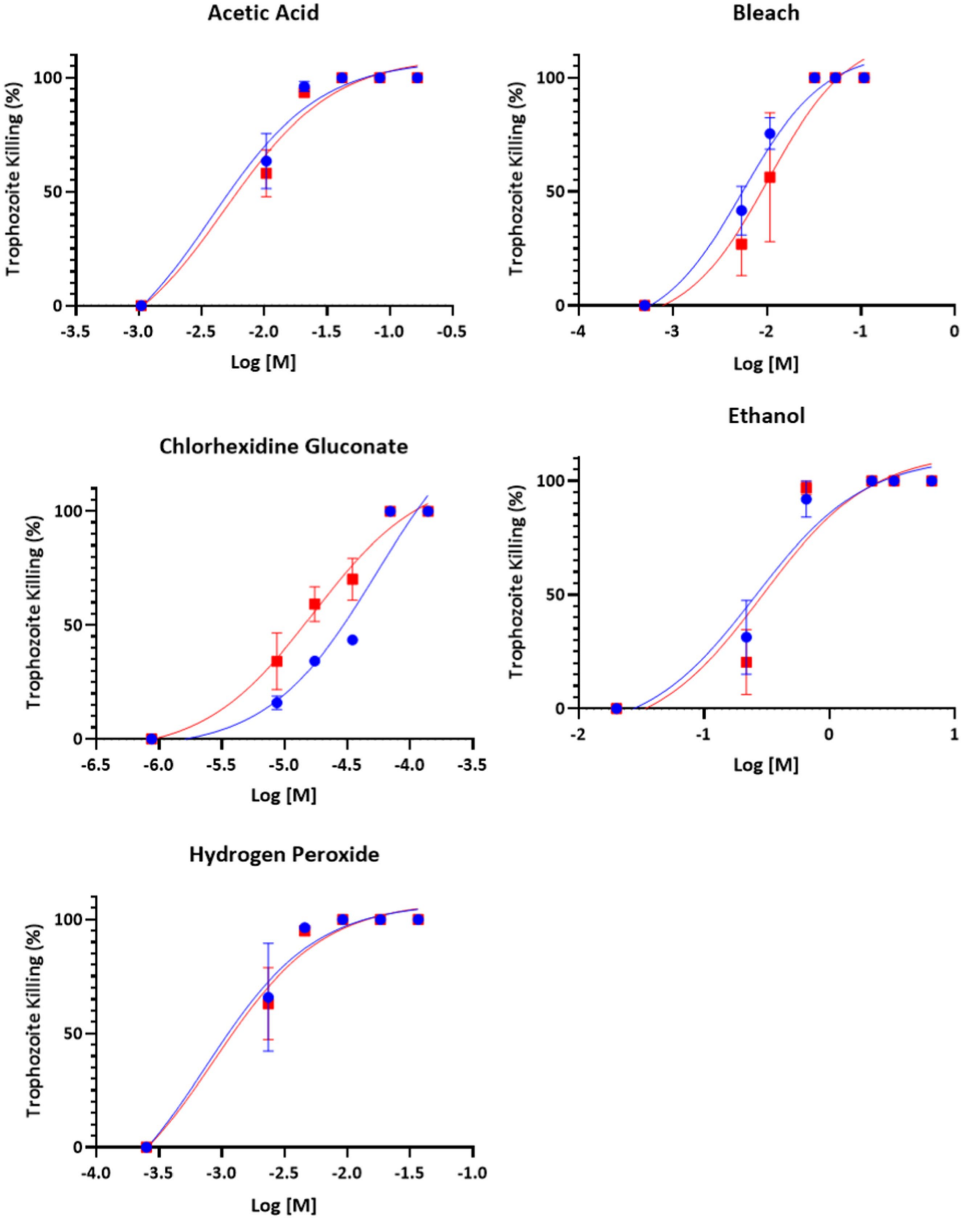
Dose–response relationship of disinfectant compounds against *T. foetus* trophozoites at 24 h *in vitro*. IA-1 trophozoites and ATCC trophozoites are represented by blue circles and red squares, respectively. Points represent mean ± SE. EC 50 values are shown in Table 3.

All compounds – acetic acid, bleach, chlorhexidine gluconate, ethanol, and hydrogen peroxide were found to be inhibitors of trophozoite growth. At 2 and 24 h, clear dose–response relationships could be observed (Figures 1, 2). Modeling of dose–response curves allowed calculation of EC_50_ values and associated 95% confidence intervals for each strain and compound combination following 2 h of treatment (Tables 2, 3). Drug free controls were included in the dose–response curves by subtracting one log unit from the lowest concentration utilized and setting the percent killing to zero since zero cannot be included on a log scale. EC_50_ values were in the micromolar range for chlorhexidine, while acetic acid, bleach, and hydrogen peroxide had EC_50_ values in the millimolar range.

**Table 2:** EC_50_ values 2 h post-treatment.

	IA-1		ATCC	
	EC_50_	95% Confidence interval	EC_50_	95% Confidence interval
Acetic acid	14.1 mM	8.8 to 22.3 mM	9.3 mM	5.3 to 15.6 mM
Bleach	28.3 mM	12.9 to 73.1 mM	28.3 mM	12.9 to 73.1 mM
Chlorhexidine gluconate	44.5 μM	23.4 to 94.9 μM	0.2 μM	* to 1.1 μM
Ethanol	0.6 M	0.2 to 2.1 M	0.6 M	0.3 to 1.4 M
Hydrogen peroxide	1.1 mM	0.6 to 1.8 mM	1.3 mM	0.8 to 1.9

Values represent mean and standard error of 3 experiments each with two technical replicates. *The lower limit of the confidence interval could not be calculated due to variability at very low concentrations.

**Table 3:** EC_50_ values at 24 h post-treatment.

	IA-1		ATCC	
	EC_50_	95% Confidence interval	EC_50_	95% Confidence interval
Acetic acid	3.8 mM	1.4 to 7.2 mM	4.9 mM	2.3 to 8.4 mM
Bleach	5.5 mM	3.3 to 9.0 mM	10.3 mM	3.7 to 31.0 mM
Chlorhexidine gluconate	54 μM	30 to 108.5 μM	17 μM	8.7 to 34 μM
Ethanol	0.3 M	0.1 to 0.5 M	0.3 M	0.1 to 0.6 M
Hydrogen peroxide	0.7 mM	** to 2.1 mM	0.9 mM	0.2 to 1.8 mM

Values represent mean and standard error of 3 experiments each with two technical replicates. **The lower limit of the confidence interval could not be calculated due to variability at very low concentrations.

### Recovery assays

**Figure 3:**
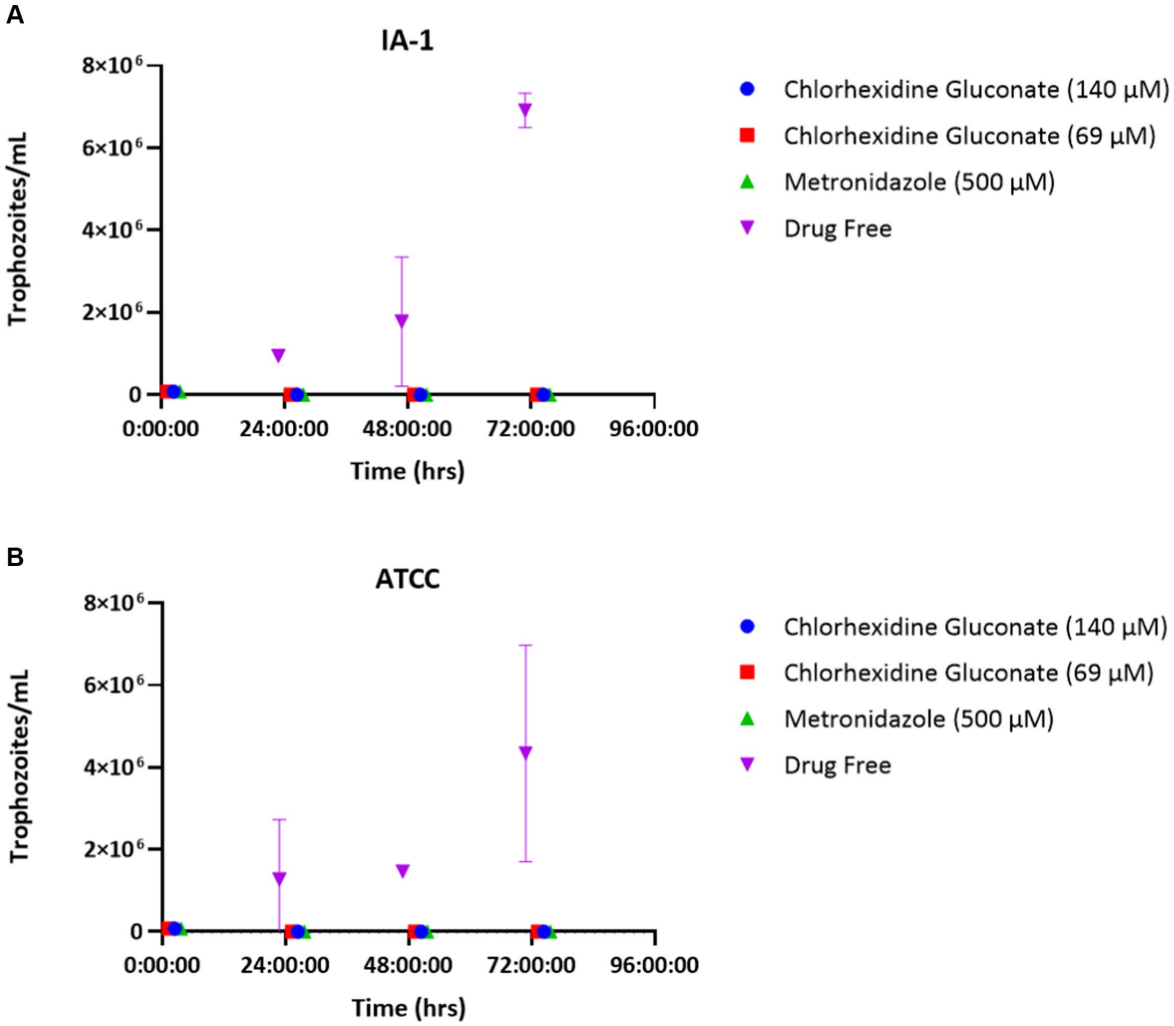
Chlorhexidine gluconate recovery assays for *T. foetus* strains IA-1 **(A)** and ATCC **(B)**. Points represent mean parasite counts ± SE.

Chlorhexidine gluconate recovery assays confirmed the *in vitro* trophocidal activity of chlorhexidine gluconate, as no trophozoites were able to recover within 72 h of drug removal for the concentrations of chlorhexidine tested (Figure 3).

### *In vitro* membrane permeability assay

**Figure 4:**
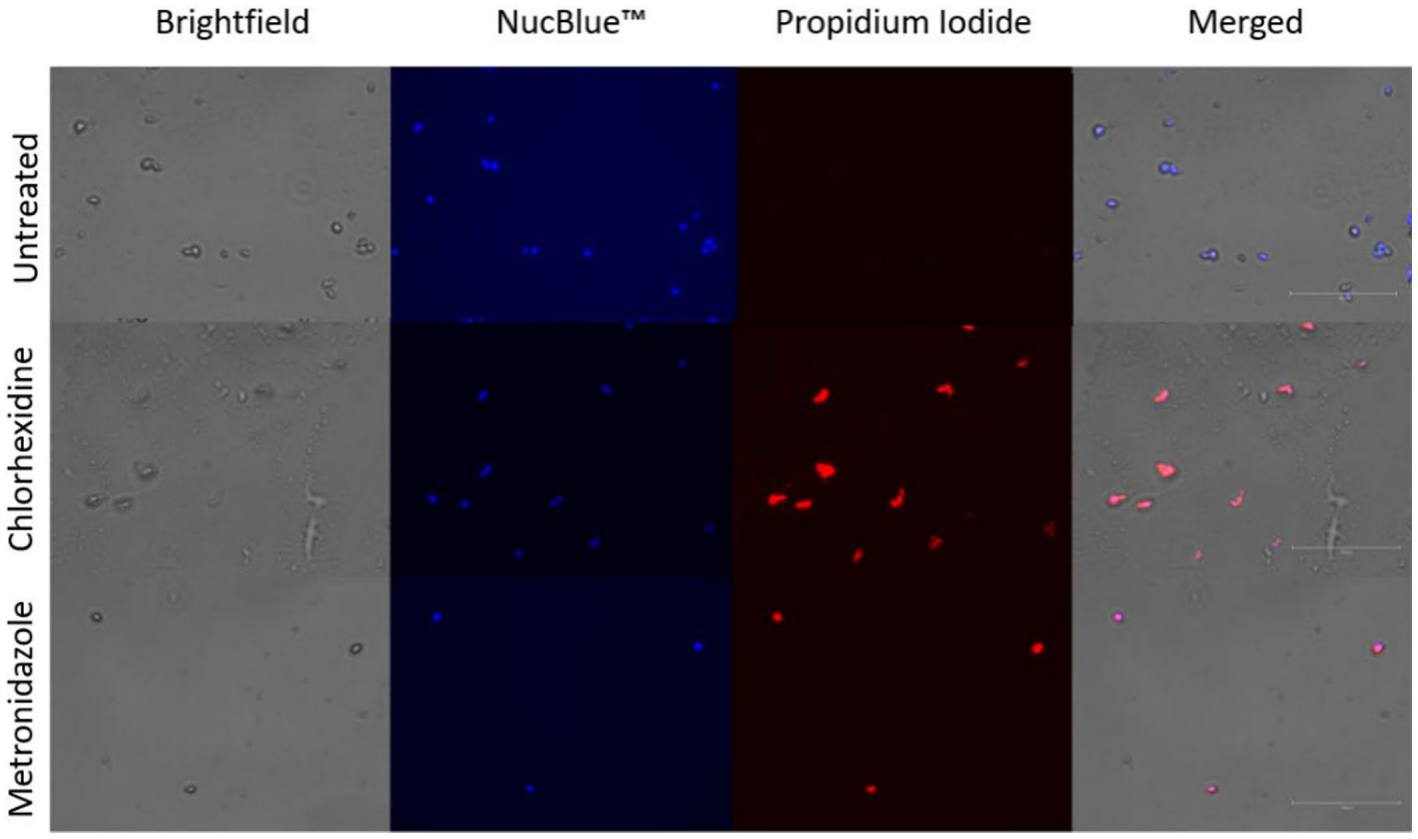
Membrane permeability assay. Trophozoites stained with NucBlue ™ and propidium iodide following co-incubation with chlorhexidine gluconate (0.05%) or metronidazole (2.5 mg/mL). Blue staining indicates nuclei, red-orange staining indicates cells with permeable membranes.

Mechanisms of action for the tested compounds are presumed to be similar for killing of other microorganisms and are not well characterized for *T. foetus*. We hypothesized that chlorhexidine, a promising compound killing trophozoites in this study, acted by detergent action that damages cell membranes. Propidium iodide was indicative of damaged, permeable trophozoite cell membranes (Figure 4).

## Discussion

This study evaluated five compounds against *T. foetus* trophozoites: acetic acid, bleach, chlorhexidine gluconate, ethanol, and hydrogen peroxide. The results provide evidence for effective surface disinfectant options for the parasite in both laboratory and clinical settings. In addition, our experiments highlighted the potential of compounds for the of treatment of bovine trichomonosis, although we did not assess this directly in infected animals. Acetic acid, chlorhexidine gluconate, and hydrogen peroxide were parasiticidal have been used on skin and mucosal surfaces for various purposes which provides support and rationale for pursuing *in vivo* treatment studies with these particular compounds (17–19).

The compounds used in this study were capable of killing trophozoites. We observed moderate differences in EC_50_ values among the strains used, which is similar to strain variability reported in other *in vitro T. foetus* treatment assays (20, 21). Further investigation assessing a broad range of *T. foetus* isolates would be favorable to ensure our observations extend across all strains, although we hypothesize that sensitivity to the disinfectant compounds used here will be widespread. Testing of additional agents, such as boric acid and povidone iodine, would also be prudent, as they have shown efficacy against *T. vaginalis* and other vaginal infections in humans (9, 10).

In terms of clinical utility and potential as an efficacious treatment of bovine trichomonosis, chlorhexidine gluconate is the most promising compound studied in this report. Chlorhexidine gluconate produced the lowest EC_50_ values for both parasite strains included in this study, and it is already utilized for many clinical purposes, including surgical site scrubs and oral rinses (22, 23). Thus, there is a reasonable expectation it can be used safely by a topical route of administration. In addition, chlorhexidine gluconate has been specifically studied for efficacy against human STIs including *Trichomonas vaginalis*, HIV, and *Neisseria gonorrhea* when delivered as a topical gel or douche (24, 25).

The combination of clinical utility, acceptance of use on mucosal surfaces, and performance in killing and recovery assays, makes chlorhexidine gluconate the top candidate from these *in vitro* studies to move forward to *in vivo* studies. It is possible a treatment as simple as a chlorhexidine flush or scrub of the penis and prepuce prior to bull turnout could stop the transmission of *T. foetus* and ultimately eliminate the parasite from beef cattle production. This treatment approach is particularly appealing because it does not involve systemic drug administration, is relatively noninvasive, and would be inexpensive. Ideally, a chute-side diagnostic test would be available to allow for targeted treatment of only infected animals.

In addition to identifying chlorhexidine gluconate as a compound worth of future study against bovine trichomonosis, this study also confirmed the activity of dilute bleach, ethanol, acetic acid, and hydrogen peroxide against trophozoites. It is important for producers and veterinarians to be aware which disinfectants are effective to reduce the chance for iatrogenic spread of *T. foetus*. Future studies can also address the ability of these compounds to on contaminated veterinary equipment.

## Data availability statement

The original contributions presented in the study are included in the article/supplementary material, further inquiries can be directed to the corresponding author.

## Author contributions

KM: Conceptualization, Investigation, Validation, Writing – original draft. KK: Investigation, Writing – review & editing. EM: Investigation, Writing – review & editing. EC: Investigation, Writing – review & editing. JJ: Conceptualization, Investigation, Writing – review & editing. MB: Writing – review & editing, Conceptualization, Funding acquisition, Resources, Supervision.
